# Genome-Wide Association Analysis for Severity of Coronary Artery Disease Using the Gensini Scoring System

**DOI:** 10.3389/fcvm.2017.00057

**Published:** 2017-09-20

**Authors:** Tanja Zeller, Moritz Seiffert, Christian Müller, Markus Scholz, Anna Schäffer, Francisco Ojeda, Heinz Drexel, Axel Mündlein, Marcus E. Kleber, Winfried März, Christoph Sinning, Fabian J. Brunner, Christoph Waldeyer, Till Keller, Christoph H. Saely, Karsten Sydow, Joachim Thiery, Daniel Teupser, Stefan Blankenberg, Renate Schnabel

**Affiliations:** ^1^Department of General and Interventional Cardiology, University Heart Center Hamburg, Hamburg, Germany; ^2^DZHK (German Centre for Cardiovascular Research), Partner Site Hamburg/Kiel/Lübeck, Hamburg, Germany; ^3^Institute for Medical Informatics, Statistics and Epidemiology, University of Leipzig, Leipzig, Germany; ^4^LIFE – Leipzig Research Center for Civilization Diseases, University of Leipzig, Leipzig, Germany; ^5^Vorarlberg Institute for Vascular Investigation and Treatment (VIVIT), Feldkirch, Austria; ^6^Private University of the Principality of Liechtenstein, Triesen, Liechtenstein; ^7^Drexel University College of Medicine, Philadelphia, PA, United States; ^8^Mannheim Medical Faculty, 5th Department of Medicine Medicine (Nephrology, Hypertensiology, Endocrinology, Diabetology, Rheumatology), Heidelberg University, Mannheim, Germany; ^9^Synlab Academy, Synlab Holding Deutschland GmbH, Mannheim, Germany; ^10^Clinical Institute of Medical and Chemical Laboratory Diagnostics, Medical University of Graz, Graz, Austria; ^11^Kerckhoff Heart and Thorax Center, Department of Cardiology, Bad Nauheim, Germany; ^12^German Centre for Cardiovascular Research (DZHK), Partner Site RheinMain, Bad Nauheim, Germany; ^13^Department of Medicine and Cardiology, Academic Teaching Hospital Feldkirch, Feldkirch, Austria; ^14^Institute of Laboratory Medicine, Clinical Chemistry and Molecular Diagnostics, University of Leipzig, Leipzig, Germany; ^15^Institute of Laboratory Medicine, University Hospital Munich (LMU) and Ludwig-Maximilian-University Munich, Munich, Germany

**Keywords:** genetics, Gensini score, polymorphism, genome-wide association, severity of coronary artery disease

## Abstract

Coronary artery disease (CAD) has a complex etiology involving numerous environmental and genetic factors of disease risk. To date, the genetic 9p21 locus represents the most robust genetic finding for prevalent and incident CAD. However, limited information is available on the genetic background of the severity and distribution of CAD. CAD manifests itself as stable CAD or acute coronary syndrome. The Gensini score quantifies the extent CAD but requires coronary angiography. Here, we aimed to identify novel genetic variants associated with Gensini score severity and distribution of CAD. A two-stage approach including a discovery and a replication stage was used to assess genetic variants. In the discovery phase, a meta-analysis of genome-wide association data of 4,930 CAD-subjects assessed by the Gensini score was performed. Selected single nucleotide polymorphisms (SNPs) were replicated in 2,283 CAD-subjects by *de novo* genotyping. We identified genetic loci located on chromosome 2 and 9 to be associated with Gensini score severity and distribution of CAD in the discovery stage. Although the loci on chromosome 2 could not be replicated in the second stage, the known CAD-locus on chromosome 9p21, represented by rs133349, was identified and, thus, was confirmed as risk locus for CAD severity.

## Introduction

Coronary artery disease (CAD) is the most common cause of death in industrialized countries, and its prevalence is rapidly increasing in developing countries. CAD has a complex and heterogeneous etiology involving numerous environmental and genetic factors of disease risk. According to epidemiological and family-based studies, the genetic heritability of CAD is suggested to be ~40–60% within a population (1). Common genetic susceptibility loci have been identified to be independently associated with CAD risk through genome-wide association studies (GWAS) (2, 3), including the 9p21 locus as being the most robust genetic finding for CAD to date. However, from a clinical perspective the observed effect sizes of these loci are small to moderate (3) which may, in part, be due to the heterogeneity of the different CAD phenotypes that were examined ranging from population at risk of developing CAD over anamnestic CAD to angiographically confirmed CAD and from stable disease to acute coronary syndrome.

In primary prevention, population risk of CAD is established mainly by the Framingham Risk Score (4). However, in recent years, this attributable risk was refined by additional markers indicating low, intermediate, or high risk populations (5). Coronary angiography remains the gold standard to assess CAD (6). The Gensini score (7) was established to quantify the severity and extent of CAD, including stenosis severity and anatomical location according to its functional relevance in the coronary circulation. An important advantage of using the Gensini score over others (e.g., SYNTAX score) is that it includes early alterations of atherosclerotic disease and mild stenoses.

In this study, we aimed to identify additional genetic loci more specific to the severity and distribution of CAD. We carried out a genome-wide approach of genetic variations with the Gensini score in European patients who underwent coronary angiography.

## Materials and Methods

### GWA Meta-analysis—Discovery

We conducted a meta-analysis of GWAS data in 4,931 participants of European ancestry from three large cohorts. The study was performed in the Athero*Gene* study (8), the LIFE Heart Study (9), and the LURIC study (10). Each study was carried out in accordance with the recommendations of the local ethics committee, and all participants gave written informed consent in accordance with the Declaration of Helsinki. The protocols were approved by the respective local ethics and data safety committee. The cohorts are described in detail in the Supplementary Material.

### Assessment of the Gensini Score

Coronary artery disease severity was assessed according to the method described by Gensini (7). Briefly, each coronary artery lesion is scored for the diameter stenosis (25% = 1, 50% = 2, 75% = 4, 90% = 8, 99% = 16, complete occlusion) This score is multiplied with a predefined factor according to the functional relevance of the diseased vascular segment. This calculation yields a sum score describing CAD severity in each patient.

### Genotyping and Imputation

Genotyping was conducted using the Affymetrix Whole-Genome Human SNP Array 6.0 in Athero*Gene* and LURIC and using the Affymetrix Axiom CADLIFE Array in LIFE Heart. Processing of DNA samples was performed using the Affymetrix Genome-Wide Human SNP Nsp/Sty Assay 5.0/6.0, and the Affymetrix/Axiom Reagent kit, respectively, and hybridization was done in accordance with the manufacturers’ standard recommendations. Details on quality control used for genotyping are described in the Supplementary Material.

Each study imputed genotype data to 2.5 million non-monomorphic, autosomal SNPs using HapMap haplotypes (CEU population, release 24, build 36) as reference. Imputation was performed with the software IMPUTE v2.1.0. as reference. Analyses were restricted to individuals of European descent only.

### Statistical Analyses

Genome-wide association study was conducted in each discovery cohort independently. All individuals with coronary artery bypasses or with a Gensini score = 0 were excluded from analysis to ensure that the observed effect was driven by the continuous severity of CAD rather than binary presence or absence of the disease. Data were analyzed by applying linear regression with an additive genetic model (1 degree of freedom trend test) to evaluate the association between log-transformed Gensini scores and genotypes (0, 1, and 2 variant alleles). Analyses were performed with adjustment for age and gender in all studies. To account for population stratification, we additionally adjusted for the first three components from principal component analysis (PCA). A fixed-effects meta-analysis was conducted by combining individual estimates of genotype effects from Athero*Gene*, LIFE Heart, and LURIC after excluding genotyped and imputed SNPs not meeting the quality control filters (Supplementary Material).

An *a priori* threshold for genome-wide significance was 5 × 10^−8^, and a *p*-value >5 × 10^−8^ but <5 × 10^−6^ was considered moderate evidence for association.

### Replication

Replication cohorts included 2,283 participants from the VIVIT, stenoCardia, and INTERCATH studies. Details about these cohorts are provided in the Supplementary Material.

For replication phase, we selected SNPs with a *p*-value <5 × 10^−6^ from the discovery meta-analysis (rs1485086, rs2376012, rs16835318, and rs17752803) on chromosome 2 and SNP rs1333049 on chromosome 9.

*De novo* genotyping for replication was performed for the five selected SNPs in VIVIT, steno*Cardia* and INTERCATH by 5′Nuclease assays using ABI genotyping assays (Applied Biosystems, Darmstadt, Germany). Genotyping was performed in 96-well plates. The Genotyping Master Mix (Applied Biosystems, Darmstadt, Germany) was used in a 7-µl total reaction volume, including 20 ng DNA per reaction. Genotypes were automatically attributed by measuring the allele-specific fluorescence on the ABI 7900HT real-time PCR System (Applied Biosystems, Darmstadt, Germany), using the SDS 2.4 software for allele discrimination (auto caller confidence interval >95%).

Association was tested by a linear regression model assuming additive allele effects in the replication studies. For single SNP replication analysis, we assumed concordant direction of effect and a *p*-value <0.05. Combined meta-analysis was repeated for the replication cohorts alone.

## Results

The characteristics of the discovery and replication cohorts are presented in Table 1. Mean age of the study participants in the discovery cohorts was 63 years (SD ± 9.8) in AtheroGene, 63.4 years (SD ± 11.3) in LIFE Heart, and 62.3 years (SD ± 10.8) in LURIC. 22, 26.6, and 31.8% of the participants, respectively, were women. The median Gensini score was 38 (18–65.5) in AtheroGene, 24 (6–49) in LIFE Heart, and 27 (5–53.5) in LURIC.

**Table 1:** Characteristics of study participants of the three discovery cohorts in the meta-analysis of genome-wide association studies of severity of coronary artery disease using the Gensini score.

	Discovery			Replication		
	AtheroGene *n* = 1,168	LIFE Heart *n* = 1,603	LURIC *n* = 1,899	Vivit *n* = 1,235	stenoCardia *n* = 490	INTERCATH *n* = 546
Age, years, mean ± SD	63 ± 9.8	63.4 ± 11.3	62.3 ± 10.8	64.2 ± 10.5	64 ± 11	69.7 ± 10.6
Women, %	257 (22)	427 (26.6)	603 (31.8)	343 (27.8)	144 (29.4)	151 (27.8)
Gensini score, median (25th–75th percentile)	38 (18–65.5)	24 (6–48)	27 (5–53.5)	15 (1–42)	23 (9–48)	16 (6.5–38)
Hypertension, %	838 (71.7)	1067 (66.6)	1364 (71.8)	1012 (82.0)	383 (78.2)	429 (81.2)
Diabetes mellitus, *N* (%)	248 (21.2)	533 (33.3)	746 (39.3)	366 (29.7)	83 (16.9)	151 (28.3)
Current cigarette smoker, *N* (%)	366 (31.3)	421 (26.3)	455 (24.0)	229 (18.6)	147 (30.1)	84 (16.5)
Former cigarette smoker, *N* (%)	363 (31.1)	624 (38.9)	723 (38.1)	535 (43.4)	140 (29.1)	249 (48.9)
Total cholesterol, mg/dL, mean ± SD	215.0 ± 48.5	207.0 ± 47.5	193.4 ± 38.9	204.7 ± 46.9	209.2 ± 47.0	167.7 ± 47.7
HDL cholesterol, mg/dL, mean ± SD	48.8 ± 14.4	47.5 ± 13.6	39.0 ± 10.9	52.6 ± 15.5	48.5 ± 13.6	47.8 ± 21.2
LDL cholesterol, mg/dL, mean ± SD	138.8 ± 43	129 ± 42	116.6 ± 34.4	128.03 ± 39.12	130.2 ± 42.2	93.6 ± 38.6
Triglycerides, mg/dL, mean ± SD	165.37 ± 114.6	173 ± 108	170.9 ± 121.6	151.6 ± 99.8	162.2 ± 123.9	153.8 ± 195.7
Body mass index, kg/m^2^, mean ± SD	27.5 ± 3.7	29.5 ± 4.8	27.5 ± 4.1	27.4 ± 4.2	27.9 ± 4.2	27.2 ± 5.2
History of MI, *N* (%)	280 (24)	400 (25)	710 (37.4)	363 (29.3)	95 (19.8)	123 (23.5)

In the replication cohorts, mean age was 64.2 years (SD ± 10.5) in Vivit, 64 years (SD ± 11) in stenoCardia, and 69.7 years (SD ± 10.6) in INTERCATH. 27.8, 29.4, and 27.8% of the participants, respectively, were women. The median Gensini score was 15 (1–42) in Vivit, 23 (9–48) in stenoCardia, and 16 (6.5–38) in INTERCATH.

**Figure 1:**
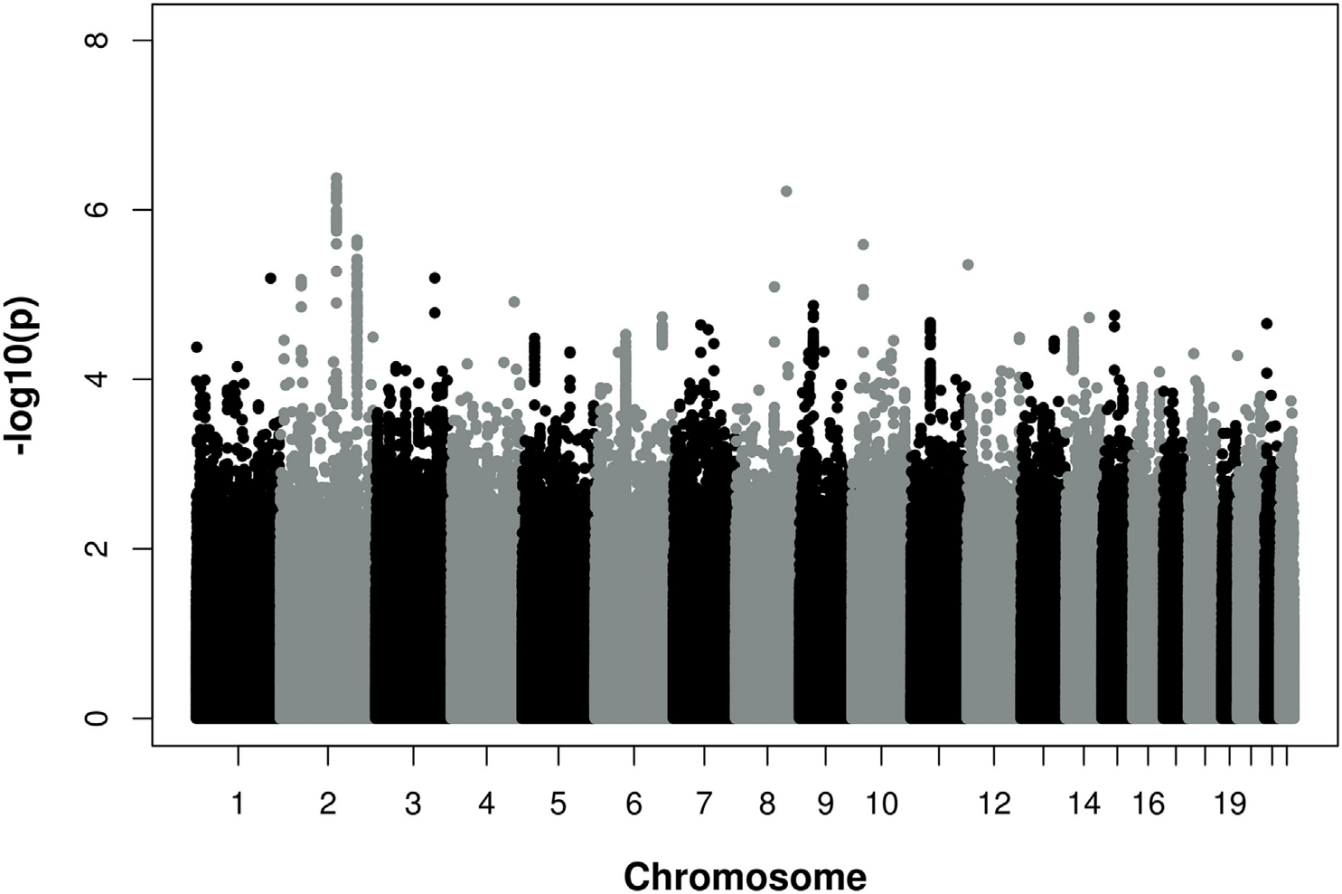
Manhattan Plot of −log10 (*p*) for association of single nucleotide polymorphisms (SNPs) and chromosomal position for all autosomal SNPs analyzed in the meta-analysis of three independent discovery cohorts. Associations with a *p*–value <5 × 10^−8^ were considered genome-wide significant. The analysis was adjusted for age and gender.

Figure 1 provides a Manhattan plot of the meta-analysis *p*-values by chromosomal positions. Results of the meta-analysis are shown in Table S1 in Supplementary Material. None of the SNPs reached genome-wide significance according to the conservative Bonferroni threshold of *P* < 5 × 10^−8^. However, two loci in an intergenic region on chromosome 2 (2q22.1 and 2q32.3) reached *p*-values of <5 × 10^−6^. The 2q22.1 region was represented by rs1485086-C [*p* = 7.8 × 10^−7^, beta = 0.196 (SD 0.04)], rs2376012-A [*p* = 7.7 × 10^−7^, beta = 0.128 (SD 0.026)], rs17752803-T [*p* = 4.2 × 10^−7^, beta = −0.214 (SD 0.042)], and 2q32.2 by rs16835318-A [*p* = 3.8 × 10^−6^, beta = 0.191 (SD 0.041)]. In addition, on chromosome 9p21, the most significant and known CAD locus so far, several SNPs showed an association with CAD severity. These SNPs included rs4977575-G [1.3 × 10^−5^, beta = 0.103 (SD 0.024)], rs1333047-T [1.7 × 10^−5^, beta = 0.102 (SD 0.024)], and rs1333049-C [1.9 × 10^−5^, beta = 0.101 (SD 0.024)].

To validate the regions on chromosome 2, the four SNPs (rs1485086, rs2376012, rs16835318, and rs17752803) were selected for *de novo* genotyping in our replication cohorts. In addition, we assessed SNP rs1333049 on chromosome 9 (as representative SNP of this known CAD locus) in our replication cohorts.

**Figure 2:**
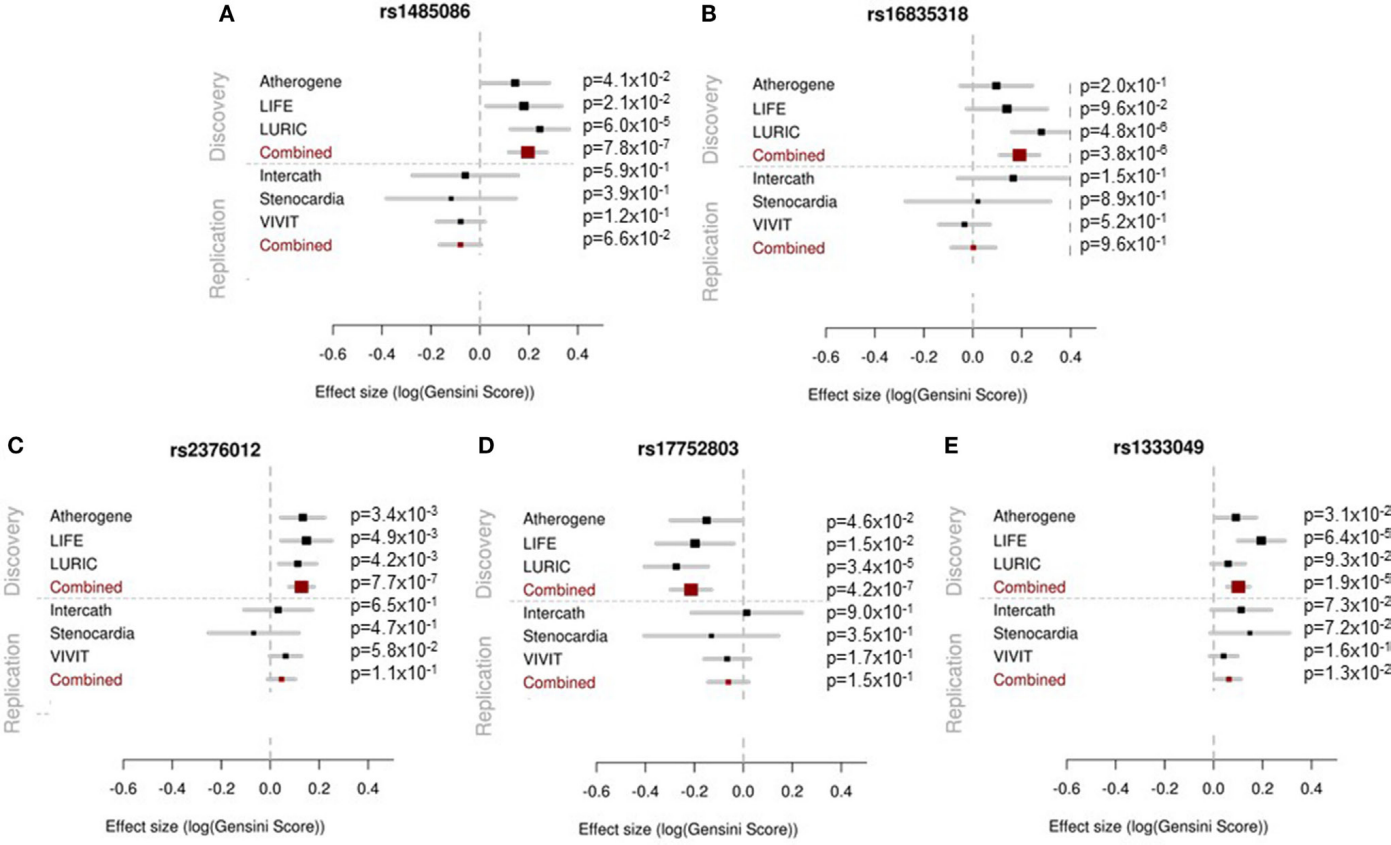
Forest plots showing the results of the association analyses for each single study in the discovery (upper panel) and replication (lower panel) phase and for the meta-analysis are presented. The effect estimates and 95% confidence intervals are provided for each study separately and in combined analysis.

None of the four SNPs on chromosome 2 reached the predefined statistical significance for replication and, thus, could not be considered as replicated neither as single SNPs nor in the combined meta-analysis. However, the locus on chromosome 9p21 reached the statistical significance threshold for replication and was confirmed as locus for the severity of stenosis in CAD [rs1333049-C, *p* = 1.27 × 10^−2^, beta = 0.062 (SD 0.025)]. The results of the replication and combined analysis are listed in Table 2 and Figure 2.

**Table 2:** Effects of selected SNPs in the combined discovery and replication phase.

							Discovery		Replication	
SNP ID	chr	Pos	Coded allele	Other allele	Nearest gene	Frequency coded allele [%]	Beta (SE)	*p*-Value	Beta (SE)	*p*-Value
rs1485086	2	139722479	C	G	Upstream of NXPH2 (184.7kb)	9.5	0.196 (0.040)	7.82 × 10*^−^*^7^	−0.079 (0.043)	6.63 × 10*^−^*^2^
rs16835318	2	194659522	A	G	–	8.8	0.191 (0.041)	3.83 × 10*^−^*^6^	0.003 (0.046)	9.56 × 10*^−^*^1^
rs2376012	2	139648059	A	G	Upstream of NXPH2 (110.2 kb)	27.4	0.128 (0.026)	7.66 × 10*^−^*^7^	0.046 (0.028)	1.07 × 10*^−^*^1^
rs17752803	2	139810489	T	C	Upstream of NXPH2 (272.7 kb)	10.0	*−*0.214 (0.042)	4.20 × 10*^−^*^7^	*−*0.061 (0.042)	1.48 × 10*^−^*^1^
rs1333049	9	22125503	C	G	Downstream of CDKN2B-AS1 (4.4kb)	49.0	0.101 (0.024)	1.86 × 10*^−^*^5^	0.062 (0.025)	1.27 × 10*^−^*^2^

*For single SNP replication analysis, we assumed concordant direction of effect and p-value <0.05*.

## Discussion

Our genome-wide association (GWA) study of angiographically defined CAD revealed that the well-established 9p21 locus is not only associated with disease risk but also severity and extent of CAD. However, this refined CAD phenotype did not lead to the identification of novel loci beyond this known locus in our medium-sized study samples.

Coronary angiography remains the gold standard for the assessment of CAD at current stage. A continuous acquisition of CAD severity may be superior to a binary assessment of CAD presence due to its progressive nature over time. Scoring systems such as the Gensini score (7) reflect the severity and extent of CAD and precisely define the disease phenotype. With the progression of CAD, an individual’s Gensini score may increase over time. Accordingly, analyses were adjusted for age of the patients. Prognostic implications of the Gensini score on overall survival have been demonstrated (11). CAD is a heterogeneous phenotype that is not fully mirrored by most of the recent GWAS (12). For example, high heritability estimates have been reported for left-main disease and calcified coronary lesions (13). The Gensini score provides a detailed representation across the spectrum of CAD as it already reflects minor angiographically detectable vascular alterations and, thus, should more accurately subclassify individuals with a diagnosis of CAD.

Recent studies have demonstrated an association between 9p21 and CAD burden with number of diseased vessels or semiquantitative methods such as the Gensini score, suggesting that 9p21 promotes progressive atherosclerosis (14–18). By contrast, other studies have not confirmed this association, and this lack of consistency has led to difficulties in reconciling association with presence but not extent of CAD (19, 20).

A recent large meta-analysis, which included a breadth of published and unpublished reports on 9p21 and angiographic CAD, convincingly demonstrated that 9p21 was associated with greater CAD burden as marker of more severe atherosclerosis but not with prevalent myocardial infarction (21).

In this study, we aimed to identify genetic loci more specifically associated with the severity and distribution of CAD by using a two-stage approach including a discovery stage and a subsequent replication stage. While none of the SNPs reached genome-wide significance in the discovery stage, several SNPs on chromosome 2 and chromosome 9 showed borderline significance. In the replication phase, the locus on chromosome 2 could not be verified; however, our results reconfirmed the known chromosome 9p21 locus as locus for CAD severity and distribution.

Strengths of this study include the availability of data from three large CAD studies, independent replication of results, and standardized definition and assessment of the extent of CAD through a validated semiquantitative score. Furthermore, similarity in quality control measures across cohorts, imputation strategies, and analytical methods account for homogeneity in analyses.

Some important limitations of our study should be noted. Compared to other GWAS samples, our combined samples were only of medium size because of the rigorous assessment of CAD that is not widely available in current GWAS studies. However, based on our statistical power, we should have been able to find medium to large effects with possible clinical relevance. Examination of larger cohorts with CAD defined by the Gensini Score may detect more genome-wide significant associations. In addition, we cannot generalize our findings to individuals of non-European ancestry. Assessment of CAD severity by semiquantitative angiographic scoring relies on angiographically detectable lesions. Disease burden that is limited to the vascular wall without significant intraluminal progression may be missed by this evaluation.

In summary, in-depth phenotyping of CAD using the angiographically determined Gensini score confirmed the chromosome 9p21 locus as risk locus of CAD severity. No additional locus of CAD severity was identified in this study. Genetic correlates of the CAD scoring systems need to be investigated in larger cohorts.

## Ethics Statement

Each study was carried out in accordance with the recommendations of the local ethics committee, and all participants gave written informed consent in accordance with the Declaration of Helsinki. The protocols were approved by the respective local ethics and data safety committee. The cohorts are described in detail in the Supplementary Material.

## Author Contributions

TZ and MoS: study design, data acquisition, writing of manuscript, and critical revision of manuscript. CM: data analyses and meta analyses, and critical revision of manuscript. MS, FO, and MK: data analyses and critical revision of manuscript. AS, HD, AM, WM, CS, FB, CW, TK, CHS, KS, JT, DT: data acquisition and revision of manuscript. SB: study design, writing of manuscript and critical revision of manuscript. RS: study design, data acquisition, writing of manuscript, and critical revision of manuscript.

## Conflict of Interest Statement

SB has received research funding from Abbott, Abbott Diagnostics, Bayer, Boehringer Ingelheim, SIEMENS, Singulex, and Thermo Fisher; honoraria for lectures from Abbott, Abbott Diagnostics, Astra Zeneca, Bayer, Boehringer Ingelheim, Medtronic, Pfizer, Roche, SIEMENS Diagnostics, SIEMENS, Thermo Fisher and as member of Advisory Boards and for consulting for Boehringer Ingelheim, Bayer, Novartis, Roche, and Thermo Fisher. The handling Editor declared a shared affiliation, though no other collaboration, with the authors MK and WM.
