# Supplementary material for: Genome-Wide Association Analysis for Severity of Coronary Artery Disease Using the Gensini Scoring System
Source: Front Cardiovasc Med. 2017 Sep 20;4:57. doi: 10.3389/fcvm.2017.00057 (PMC5611399; doi:10.3389/fcvm.2017.00057)
Supplement: Supplementary file 2 [file data_sheet_1.docx]

**Supplementary Material**

**Genome-wide association analysis for severity of coronary artery disease using the Gensini scoring system**

Tanja Zeller ^1,2*^, Moritz Seiffert ^1,2*^, Christian Müller ^1,2^, Markus Scholz ^3,4^, Anna Schäffer ^1^, Francisco Ojeda ^1^, Heinz Drexel ^5,6,7^, Axel Mündlein ^5^, Marcus E Kleber ^8^, Winfried März ^8,9,10^, Christoph Sinning ^1^, Fabian J Brunner ^1^, Christoph Waldeyer ^1^, Till Keller ^11,12^, Christoph H Saely ^5,6,13^, Karsten Sydow ^1^, Joachim Thiery ^4,14^, Daniel Teupser ^4,15^, Stefan Blankenberg ^1,2^, Renate Schnabel ^1,2^

1. Department of General and Interventional Cardiology, University Heart Center Hamburg, Martinistraße 52, 20246 Hamburg, Germany
2. DZHK (German Centre for Cardiovascular Research), partner site Hamburg/Kiel/Lübeck, Hamburg, Germany
3. Institute for Medical Informatics, Statistics and Epidemiology, University of Leipzig, Germany.
4. LIFE – Leipzig Research Center for Civilization Diseases, University of Leipzig, Germany
5. Vorarlberg Institute for Vascular Investigation and Treatment (VIVIT), Feldkirch, Austria
6. Private University of the Principality of Liechtenstein, Triesen, Liechtenstein
7. Drexel University College of Medicine, Philadelphia, PA, USA
8. 5th Department of Medicine Medicine (Nephrology, Hypertensiology, Endocrinology, Diabetology, Rheumatology), Mannheim Medical Faculty, University of Heidelberg, Mannheim, Germany
9. Synlab Academy, Synlab Holding Deutschland GmbH, Mannheim, Germany
10. Clinical Institute of Medical and Chemical Laboratory Diagnostics, Medical University of Graz, Graz, Austria
11. Kerckhoff Heart and Thorax Center, Department of Cardiology, Bad Nauheim, Germany
12. German Centre for Cardiovascular Research (DZHK), partner site RheinMain, Bad Nauheim, Germany
13. Department of Medicine and Cardiology, Academic Teaching Hospital Feldkirch, Feldkirch, Austria
14. Institute of Laboratory Medicine, Clinical Chemistry and Molecular Diagnostics, University of Leipzig, Germany.
15. Institute of Laboratory Medicine, University Hospital Munich (LMU) and Ludwig-Maximilian- University Munich, Germany.

Corresponding author: t.zeller@uke.de

**Supplementary Material**

**1. Description of study cohorts**

**2. Quality control of genotyping data**

**3. SNP inclusion criteria for meta-analysis**

**Supplementary Tables**

**Supplementary Tables 1: Results from genome-wide association analysis.**

1. **Description of study cohorts**

**Discovery**

***AtheroGene***

The Athero*Gene* study is a cohort of patients with documented coronary heart disease enrolled at the Johannes Gutenberg University, Mainz, Germany and Bundeswehrzentralkrankenhaus, Koblenz, Germany who have been followed up for cardiovascular death by questionnaire and telephone interview over a median of 4.9 (maximum 7.6) years ([Blankenberg et al., 2003](#_ENREF_2)). The study was approved by the local ethics committees. Participation was voluntary; each patient gave written, informed consent.

***LIFE Heart***

LIFE Heart is a cohort of patients with suspected coronary artery disease, stable coronary artery disease or myocardial infarction collected at the Heart Center Leipzig, Germany. All patients underwent coronary-angiography. Details of the study can be found elsewhere ([Beutner et al., 2011](#_ENREF_1)). Individuals were genotyped using the Affymetrix Axiom Technology with custom option (Axiom-CADLIFE). Genotypes were imputed to HapMap2 reference (release 24, built 36) using IMPUTE v2.1.2.

The study has been approved by the Ethics Committee of the Medical Faculty of the University Leipzig, Germany (Reg. No 276-2005) and is registered at ClinicalTrials.gov (NCT00497887). Written informed consent was obtained from all participants.

***LURIC***

The Ludwigshafen Risk and Cardiovascular Health (LURIC) study is a monocentric hospital based prospective study including 3316 individuals referred for coronary angiography recruited in the Ludwigshafen Cardiac Center, southwestern Germany from 1997 – 2000 ([Winkelmann et al., 2001](#_ENREF_6)). Clinical indications for angiography were chest pain or a positive non-invasive stress test suggestive of myocardial ischemia. To limit clinical heterogeneity, individuals suffering from acute illnesses other than acute coronary syndrome, chronic non-cardiac diseases and a history of malignancy within the five past years were excluded. All participants were completed a detailed questionnaire which gathered information on medical history, clinical, and lifestyle factors. Fasting blood samples were obtained by venipuncture in the early morning and stored for later analyses. The ethics committee at the "Landesärztekammer Rheinland-Pfalz" approved the study and informed written consent was obtained from all participants.

**Replication**

***VIVIT***

The VIVIT study included two cohorts of consecutive patients undergoing coronary angiography for the evaluation of suspected or established stable coronary artery disease from September 1999 through October 2000 and from August 2005 through December 2007, respectively, at the Department of Medicine and Cardiology of the Academic Teaching Hospital Feldkirch, Austria. Details on the recruitment protocol and the determination of subject characteristics have been reported previously ([Muendlein et al., 2009](#_ENREF_5)). The ethics committee of the University of Innsbruck approved the presented study, and all participants gave written informed consent.

***stenoCardia***

Patients with acute chest pain presenting consecutively at the chest pain unit of the Johannes Gutenberg-University Medical Centre Mainz between January 2007 and December 2008 were enrolled in this all-comers prospective biomarker assessment registry as described earlier ([Keller et al., 2009](#_ENREF_4)). Genomic DNA was extracted from buffy coat of EDTA blood and stored at -80°C. The study was approved by the local ethics committees. Participation was voluntary; each patient gave written, informed consent.

***INTERCATH***

The INTERCATH study is an ongoing contemporary cohort of patients undergoing invasive coronary angiography at the University Heart Center Hamburg, Germany. Each patient provides a dedicated questionnaire including past medical history, cardiovascular risk factors and symptoms in addition to standard laboratory values and material for biobanking. Consecutive patients from 01/2015 to 12/2015 were included in this analysis. Patients with previous coronary artery bypass grafting were excluded from this analysis due to the absence of valid coronary artery disease (CAD) scoring systems in those patients, leaving a total of n=546 cases for evaluation.

1. **Quality control of genotyping data**

**AtheroGene**

Genotyping was performed using the Affymetrix Genome-Wide Human SNP Array 6.0 as described by the Affymetrix user manual. Genotypes were called using the Affymetrix Birdseed v2 calling algorithm. Individuals with a call rate <97%, an autosomal heterozygosity >3 SDs around the mean, or a cryptic relatedness > 0.125 were excluded. Standard quality criteria were applied to exclude SNPs (MAF<1%, genotype call rate <98%, and P value of deviation from HWE <10^-4^). Imputation based on HapMap2 (NCBI Build 36) was performed using IMPUTE2. In total, 1,168 individuals with genotype data and Gensini scores > 0 were available for analysis.

**LURIC**

Genotyping was performed using the Affymetrix Genome-Wide Human SNP Array 6.0 as described by the Affymetrix user manual. Genotypes were called using the Affymetrix Birdseed v2 calling algorithm. Individuals with a call rate <95%, or cryptic relatedness were excluded. Standard quality criteria were applied to exclude SNPs (MAF<1%, genotype call rate <98%, and P value of deviation from HWE <10^-4^). Imputation based on HapMap2 (NCBI Build 36) was performed using IMPUTE2

**LIFE Heart**

Genotyping was performed at Affymetrix (Santa Clara, Ca; USA) using the Axiom technology with custom content (Axiom-CADLIFE). Affymetrix power tools version 1.12 were used for genotype calling. Sample quality criteria comprised sex mismatch, call rate<97%, low or high mean squared difference of individual’s genotype and expected genotype according to box plot outlier criteria, duplicates, implausible relatedness and outliers of principal components analysis. SNP quality criteria comprised minimal plate-wise call rate<90%, p-value of asymptotic Hardy-Weinberg equilibrium test <1.0x10^-6^, and, p-value of the association of SNP allele frequency with plate number <1.0x10^-7^.

Genotype imputation was performed using IMPUTE v2.1.2. HapMap2 CEU, Release 24, dbSNP-build 126, NCBI built 36 served as reference panel. Details of genotyping and imputation are described in ([Burkhardt et al., 2015](#_ENREF_3))

1. **SNP inclusion criteria for meta-analysis**

Only SNPs with a minor allele frequency (MAF) ≥ 5% and an imputation quality > 0.7 (IMPUTE info score) were included in the meta-analysis.

References

Beutner, F., Teupser, D., Gielen, S., Holdt, L.M., Scholz, M., Boudriot, E., et al. (2011). Rationale and design of the Leipzig (LIFE) Heart Study: phenotyping and cardiovascular characteristics of patients with coronary artery disease. *PLoS One* 6**,** e29070. doi:10.1371/journal.pone.0029070

Blankenberg, S., Rupprecht, H.J., Bickel, C., Torzewski, M., Hafner, G., Tiret, L., et al. (2003). Glutathione peroxidase 1 activity and cardiovascular events in patients with coronary artery disease. *N Engl J Med* 349**,** 1605-1613. doi:10.1056/NEJMoa030535

Burkhardt, R., Kirsten, H., Beutner, F., Holdt, L.M., Gross, A., Teren, A., et al. (2015). Integration of Genome-Wide SNP Data and Gene-Expression Profiles Reveals Six Novel Loci and Regulatory Mechanisms for Amino Acids and Acylcarnitines in Whole Blood. *PLoS Genet* 11**,** e1005510. doi:10.1371/journal.pgen.1005510

Keller, T., Zeller, T., Peetz, D., Tzikas, S., Roth, A., Czyz, E., et al. (2009). Sensitive troponin I assay in early diagnosis of acute myocardial infarction. *N Engl J Med* 361**,** 868-877. doi:10.1056/NEJMoa0903515

Muendlein, A., Saely, C.H., Rhomberg, S., Sonderegger, G., Loacker, S., Rein, P., et al. (2009). Evaluation of the association of genetic variants on the chromosomal loci 9p21.3, 6q25.1, and 2q36.3 with angiographically characterized coronary artery disease. *Atherosclerosis* 205**,** 174-180. doi:10.1016/j.atherosclerosis.2008.10.035

Winkelmann, B.R., Marz, W., Boehm, B.O., Zotz, R., Hager, J., Hellstern, P., et al. (2001). Rationale and design of the LURIC study--a resource for functional genomics, pharmacogenomics and long-term prognosis of cardiovascular disease. *Pharmacogenomics* 2**,** S1-73. doi:10.1517/14622416.2.1.S1
